# Exploring Use of Nontraditional Tobacco Products Through Focus Groups with Young Adult Smokers, 2002

**Published:** 2008-06-15

**Authors:** Patricia Richter, Ralph Caraballo, Linda L Pederson, Nisha Gupta

**Affiliations:** Office on Smoking and Health, National Center for Chronic Disease Prevention and Health Promotion, Centers for Disease Control and Prevention. Dr Pederson is now an independent consultant in Atlanta; Office on Smoking and Health, Centers for Disease Control and Prevention, Atlanta, Georgia; Office on Smoking and Health, Centers for Disease Control and Prevention, Atlanta, Georgia; Global AIDS Program, Centers for Disease Control and Prevention, and the Consulate General of the United States of America, Chennai, India

## Abstract

**Introduction:**

In 2002, 16 focus groups with young adult smokers who used or had tried nontraditional tobacco products (e.g., bidis, shisha, herbal cigarettes, kreteks, cigars, herbal smokeless products) were conducted in Dallas, Texas, and Chattanooga, Tennessee, to gain an understanding of the appeal of these products.

**Methods:**

In each city, groups were segmented by race or ethnicity and by educational status.

**Results:**

Many consistent themes emerged across the groups. Nontraditional tobacco use is not common among young adult smokers. Although some products such as Black & Mild and Swisher Sweets cigars are used frequently by some groups, other products such as shisha, kreteks, and herbal cigarettes are less well known and infrequently used. Among focus group participants, use of nontraditional tobacco products tends to occur in clubs, during social gatherings, or at times when cigarettes are unavailable. More college students than those who were not in college cited cost and inconvenience of purchasing nontraditional tobacco products as reasons for not using them. All focus group participants agreed that African Americans use cigars more than any other racial or ethnic group.

**Conclusion:**

Overall, findings suggest that the reasons for trying nontraditional tobacco products did not differ by race or ethnicity. Family members and peers were mentioned as the source of nontraditional tobacco products when first used. Cost, convenience, taste, smell, and strength were given as reasons both for using these products and for discontinuing their use.

## Introduction

Several noncigarette tobacco and herbal products are available to adults in the United States, and they are collectively referred to in this article as nontraditional tobacco products (NTPs). These products are bidis, shisha (water pipe, hookah, or hubble bubble), herbal cigarettes, kreteks (clove cigarettes), cigars (e.g., Swisher Sweets, Black & Mild), and herbal smokeless products (snuff or chew); they are described in more detail in the Appendix. The possibility that some of these products have fewer deleterious health effects than traditional cigarettes has recently been discussed (1), although there is not enough evidence yet to prove or disprove this notion. Recent research shows that young adult smokers perceive many NTPs to be safer than cigarettes (2).

In 2004, 23.6% of U.S. adults aged 18 to 24 years (6.9 million) were cigarette smokers (3). There are noted differences in smoking prevalence for young adults who are college students and those who are not. Those 18- to 22-year-olds enrolled full time in college in 2002 were less likely to report current cigarette use than their peers who were not enrolled full time, and past-month cigarette use was reported by 33% of full-time college students (ages 18–22), compared with 46% of their unenrolled peers (4). Sixty percent of college students tried cigarette smoking and more than 28% of college students report that they are current smokers (3). However, traditional cigarettes are not the only tobacco product used by young U.S. adults: in 2001, according to the National Household Survey on Drug Use and Health, 11.4% (3.3 million) used cigars, 4.7% (1.4 million) used smokeless tobacco, and 0.9% (0.3 million) used pipes (4). However, some tobacco users used more than 1 type of tobacco product. Rigotti et al (5) found that more than 50% of college students who used tobacco used more than 1 type of tobacco product, with men (58.2%) using multiple products more often than women (27.5%).

We could find little information on why smokers start using NTPs or why they continue using these products. Use of NTPs by young adults may lead to use of traditional cigarettes. Given the variety of NTPs on the market, it is important to understand the characteristics of NTP users; their perceptions about the health effects of the products; and the reasons they try, use, and discontinue use of NTPs. To learn the appeal of these products and to understand factors related to their use, we arranged for focus groups with young adult smokers who used or had tried NTPs.

## Methods

In April 2002, 16 focus groups were conducted with smokers aged 18–22 years who smoked or who had tried NTPs. Focus group participants were men and women divided into 2 groups by level of education: 1) those attending a 2- or 4-year college and 2) those not in college. The not-in-college participants were not enrolled in any school, not working on a general equivalency degree (GED), not the recipient of a GED within the previous year, and not planning to attend college during the next year. This study was approved by the Internal Review Board of the Centers for Disease Control and Prevention (CDC).

Eight focus groups were conducted in Chattanooga, Tennessee, and another 8 in Dallas, Texas. In Chattanooga, the groups were segmented by level of education and race (African American or white). In Dallas, the same segmentation was used for level of education; however, the Dallas groups were segmented by race/ethnicity (Hispanic and white). There were about equal numbers of men and women in each group.

### Recruitment

Staff at professional focus group facilities recruited participants in both cities. In Chattanooga, white and African American participants were recruited by telephone from the focus group facility's database, which included information on potential participants' age. In Dallas, white and Hispanic participants in the focus group facility's database were screened for eligibility and recruited by e-mail or telephone. In Dallas, the facility also advertised in campus newspapers for Hispanic students for the focus groups.

Recruitment screening included questions about current smoking and the use of NTPs. Current smokers were defined as those who had smoked a cigarette during the previous 30 days, which is consistent with the definitions of current smokers used by other studies (e.g., Youth Risk Behavior Survey [6], National Youth Tobacco Survey [7]). Current cigarette smokers were recruited because most researchers believe that cigarette smokers are more likely than nonsmokers to have experience with other tobacco and nontobacco products and because we were interested in how NTP use relates to the use of traditional cigarettes. Triers or users of NTPs were those who reported that they had tried or were currently using at least 1 of 6 NTPs  (Appendix). To ensure that the people who had only tried a product were not overrepresented in a group, 30% of each discussion group had to consist of current users of at least 1 NTP. To ensure that several products could be discussed, no more than 30% of the participants in each group could have tried or used only 1 of the products.

Recruitment statistics, including the total number who were screened in each city and the number who were eligible and agreed to participate, are in Table 1. In total, 137 young adults — 69 college smokers and 68 not-in-college smokers — participated in the focus groups. By race or ethnicity, they were divided into 3 groups: 70 non-Hispanic whites, 36 African Americans, and 31 Hispanics.

### Content of the sessions

On arrival at the focus group facility, participants provided demographic data and described their use of cigarettes and NTPs on a prediscussion information sheet. The focus groups, which lasted about 2 hours, were conducted in professional focus group facilities equipped with one-way mirrors, observer viewing rooms, client waiting areas, and videotape and audiotape equipment. The participants were informed about confidentiality and signed a consent form before the discussions began. They were also told that the discussion would be audiotaped and videotaped to ensure an accurate record. Participants each received $50.00 and, if they arrived at least 15 minutes before the scheduled session time, were entered into a raffle for an early bird prize of $25.

The focus group guide and questions were divided into 7 sections. The first section consisted of 1) introducing the facilitator and group members to each other and 2) discussing the topic and purpose of the focus group. Section 2 consisted of questions about cigarette smoking behavior. Sections 3 through 6 concentrated on NTPs and included discussion of personal use, use by friends and family, health perceptions about NTP use, and other perceptions about NTP use. During section 7, participants were asked to design health messages that they would use to communicate about NTPs with other people their own age.

### Data analysis

The purpose of the analysis was to uncover common and consistent themes and to note where any differences appeared between or among population segments. Two trained coders independently examined the transcripts and applied codes (1- or 2-word interpretive descriptions) to statements in the transcripts to develop categories (a group or class of related codes). The categories were then cross-classified to find relationships among them and to detect the patterns of differences or similarities. The coders discussed inconsistencies in coding until they reached an agreement on the most appropriate code. QSR NUD*IST 4 software (QSR International, Bakersfield, California, 1991) was used to organize and manage the text data and to support the coding process by allowing the text to be marked for search, retrieval, classification, and cross-classification.

## Results

### Summary of participants' characteristics and their cigarette and NTP use

Most not-in-college participants (66.1%) had either a high school diploma or a GED, and 67.6% were employed either full- or part-time. Almost 60% of the college students were either in their first or second year. Daily cigarette smoking was highest among white participants: more than 70% reported that they smoked every day during the previous month. Of participants in college, 70% smoked on fewer than 20 days per month, whereas only 40% of the not-in-college participants smoked on fewer than 20 days per month. Swisher Sweets and Black & Milds were used most often by all participants, and shisha and kreteks were used least often. None of the not-in-college participants reported having used kreteks, and only 1 reported having used a shisha. More than 50% of the participants reported having first tried an NTP at age 14–17. Table 2 shows the breakdown, by group and by NTP product, of those who reported they had used a study NTP even once.

Many of the comments about trial, use, and discontinuance of NTPs were similar for all products; therefore, the findings are organized around those 3 factors rather than by product. Most of the discussion was on bidis, kreteks, Black & Milds, and Swisher Sweets. Several participants also described blunts (marijuana mixed with tobacco in a cigar shell) when discussing cigar use (8,9).

### Why are young adults trying NTPs?

Several factors were frequently mentioned when participants were asked why they tried NTPs. Among them were social and family influences, substitution for traditional cigarettes, and curiosity about the products and their taste and smell.

#### Social and family influences

Across groups, participants said their first experiences with NTPs occurred when they were with peers or family members. Several participants reported that a friend offered them their first NTP in a social setting or that they "bummed a hit" from a friend's NTP. African Americans frequently talked about being with family members such as aunts, uncles, and cousins when they first tried these products.

#### Substitute for cigarettes

Some participants in most groups reported that they tried NTPs when cigarettes were not available.

Actually I think [I tried them] because I was out of cigarettes and all my friends were smoking [Black & Milds]. (white, not in college)

#### Curiosity

Participants in all groups reported that curiosity about the way NTPs looked or smelled motivated them to try the products. In addition, some participants mentioned being curious about the products after hearing their friends talk about how the products made them feel.

There were, like, some white boys that used to live by me that used to smoke those [bidis and kreteks]. They smelled funny, so I just wanted to see what they tasted like. (African American, college)

### Why keep using them?

Several questions were asked to learn why participants continued to use NTPs and which products they used. Most of the discussion focused on Black & Milds and Swisher Sweets. Some participants in the African American and Hispanic groups acknowledged using Black & Milds and Swisher Sweets regularly or out of habit. Some also reported that they continued to smoke kreteks, but few continued using the other products. Several reasons were cited for continuing to use NTPs: recreation, pleasant smell or taste, low cost, convenience, duration (they last longer than traditional cigarettes), and pharmacologic effects (they provide a sensation of being high and intensify the effects of other drugs such as alcohol or marijuana). Participants did not give fewer health risks as a reason for continuing to use NTPs.

#### Strength and duration

NTPs perceived by participants as "strong" were cigars and clove cigarettes. Smoking these products resulted in "getting more" than from a traditional cigarette. Cigars and clove cigarettes were noted to last longer than traditional cigarettes.

If I have a Black [& Mild] and a full pack of cigarettes, I will smoke the last Black [& Mild] before I open that pack of cigarettes, because you just get more from smoking the Black [& Mild] than from smoking a cigarette. (African American, college)

#### Cost and convenience

Several participants commented that they were able to purchase singles of an NTP rather than a pack. The ability to smoke an NTP in several sessions by extinguishing it and relighting it was also seen as a convenience and a reason to continue using the product.

[Cigars are] cheaper. It's not like a cigarette, you can buy just 1. (African American, college)

They smoke half of it [Black & Mild] and then put it out and smoke the other half later.  . . . Cloves are like that, too. (white, college)

#### Occasional or recreational use

Although daily use of NTPs appeared to be rare among participants, NTP use often occurred at parties, in clubs, and on special occasions. Some participants in the Hispanic and African American not-in-college groups discussed smoking cigars (Black & Milds and Swisher Sweets) when socializing, but they did not use them daily.

If I'm out with my boy at his apartment, we smoke Blacks [Black & Milds] because it's something you can kind of pass around to everybody and just talk. If I'm at a club or something, just me and a girl, we smoke a cigarette. (African American, not in college)

#### Taste or smell

All groups mentioned a pleasant taste or smell as reasons for using the products and said that smoking these products did not leave the smokers smelling of cigarettes.

[Bidis] taste and they smell good, so it's more like incense that sticks to you rather than tar. (white, college)

### Perceived pharmacologic effects

Some participants in all groups stated that smoking traditional cigarettes gave them a buzz or made them feel high. Their responses when discussing NTPs suggest that they look for the same sensation when using NTPs. Some participants mentioned that they tried bidis because they heard from others that they were like marijuana, but they were disappointed because, although they felt an initial high, they eventually ended up with a severe headache. In contrast, the pharmacologic effects from smoking cigars were almost uniformly described as pleasant.

It [a bidi] gave me a head rush. I was high for a little while, but after a while I started getting a headache. A nasty headache. (African American, not in college)

And [if] you smoke them [Black & Milds] fast enough, you will get a little buzz on them. (African American, college)

Participants in all groups described smoking traditional cigarettes while drinking alcohol and mentioned that NTPs enhanced the effects of alcohol.

I like Swisher Sweets and Black & Milds. I think they are a little bit smoother, but for me they are stronger. I only smoke them when I drink. . . . It kind of intensifies everything else. (Hispanic, college)

### Alternate uses of NTPs

Every group had reports of using marijuana in combination with cigars. Marijuana was spontaneously suggested as an NTP by several participants in every group. Some participants described splitting open the cigars, removing the tobacco, and replacing the tobacco with marijuana. Other participants described using NTPs to enhance the effects of marijuana.

I've smoked marijuana in them but I haven't ever smoked just a Black & Mild. (white, college)

## Why stop using NTPs?

Participants who had tried the NTPs but did not continue to use them were asked why they stopped using them and what they did not like about the products. Participants who occasionally used NTPs contributed to the discussion. Interestingly, in all groups, the reasons cited for discontinuing use were the same as the reasons given for using the products: strength, smell, taste, and cost. In addition, convenience was given as a reason for using NTPs and inconvenience as a reason for not using them. For white college groups, strength of the products played a role in discontinuing use. For African American not-in college groups, preference for cigarettes was cited as a reason for discontinuing. Hispanic participants frequently reported that they did not like the smell of NTPs.

### Strength

At least some participants in almost every group said they did not like NTPs or discontinued using them because they were too mild, and others said they were too strong or harsh. For the African American and Hispanic college groups, strength was not commonly given as a reason for discontinuing use. Some participants in the not-in-college groups also described having adverse reactions to the products, such as headaches and lightheadedness, in part because the product was too strong.

They [herbal cigarettes] were too mild so they didn't cut it anymore. (Hispanic, not in college)

[Herbal cigarettes] are too strong. (white, college)

I used to smoke [Black & Milds] . . . but it makes my head hurt and my stomach hurt. They are too strong for me. (Hispanic, not in college)

### Cost and inconvenience

White and African American college students were more likely to view the cost and inconvenience of purchasing NTPs as reasons for not using the products than were their noncollege counterparts. In every group, some people commented that the products were difficult to locate and therefore were inconvenient to use. In some groups, cigars were described as inconvenient because of the length of time it takes to smoke them.

Well, those cloves are pretty expensive, and you can only buy them from the hours of 9 to 7 at Tobacco Mart, so I decided to try to get the same effect in a shorter time. So I know cloves last 4 times as long as a regular cigarette does, so I just started smoking cigarettes instead. (white, college)

### Smell and taste

In most groups, several participants reported that they had stopped using a product because they did not like the way it smelled or tasted.

They [herbal cigarettes] smell really bad. They smell like tea bags on fire, and they taste about the same way. (white, college)

## Who are the perceived users of NTPs?

Participants were asked if they thought the use of these products differed among racial or ethnic groups, between men and women, or between people who were in college and those who were not. There was general agreement that African Americans were more likely than other groups to smoke cigars. Some African American college students said that NTP use was more common among college students because they tended to experiment more with different products. White and Hispanic groups agreed that men were more likely than women to use NTPs. Although the discussion guide developed for the groups did not ask any specific questions about age group differences, participants in the white groups frequently mentioned that NTP use was more common among teenagers than among adults, even young adults. They predicted that these young smokers would outgrow the products and graduate to traditional cigarettes eventually.

I think when you're younger is when you try it, but you grow out of it. You're just like, go to the gas station, get your pack of cigarettes, go home, smoke them, same thing tomorrow. (white, not in college)

## Discussion

Many common themes emerged across the groups. First, it became clear that continued regular use of NTPs was not common among the young adults who participated in our focus groups. Although some products, such as Black & Milds and Swisher Sweets, are widely used, other products such as shishas, kreteks, and herbal cigarettes are less well known and infrequently used. All groups agreed that cigar use was more common among African Americans. Overall, there were no apparent racial or ethnic differences in reasons for trying nontraditional products. Family members and peers were listed as sources of initial NTP use for all groups. Taste, smell, cost, and strength of the products were given as both reasons for using NTPs and for discontinuing use. Convenience was given as a reason for using NTPs, and inconvenience as a reason for not using them. Most focus group participants used NTPs at clubs, during other social gatherings, or as an alternative to cigarettes. Even when participants reported using NTPs more than occasionally, they indicated that their NTP use (most commonly cigar use) often coincided with special activities or events such as parties with friends.

However, some differences between the groups were noted. College participants were more likely than those who were not in college to cite the cost and inconvenience of purchasing NTPs as reasons for not using them. Whites were more likely to discuss experimenting with NTPs as a gateway to regular cigarette use.

These findings should be interpreted with caution because of some limitations in the methods. The focus groups were conducted in 2 midsized American cities, and the findings may not be generalizable to other larger or smaller urban areas or to rural areas. In addition, because of limited availability of some NTPs (such as shishas or kreteks), some participants did not have experience with those products, and hence, discussion about them was somewhat hampered. The focus groups were conducted in 2002, so the findings may not be relevant to the current situation. Our results are similar to survey data from Massachusetts in which approximately 1 in 5 adolescents reported that they liked the taste and smell of cigars, bidis, and kreteks, and these products gave them something novel to try (10). Other limitations include reliance on self-reported measures of smoking behavior and NTP use and the possibility that the participants withheld information or provided incomplete or misleading information. Finally, as with all focus groups, the tone and content of the discussions may have been affected by group dynamics or by the participants' knowledge that they were being audiotaped and videotaped.

Strengths of the study include the following:

- Each focus group was conducted in duplicate (i.e., 2 focus groups were conducted with each racial or ethnic group of college smokers, and 2 were conducted with each racial or ethnic group of not-in-college smokers). This strategy allowed us to determine the replicability of participants' experiences and opinions.
- The recruiting strategy allowed each group to include participants who had tried a variety of the products.
- We were able to investigate factors related to trial, use, and discontinued use of NTPs by different racial or ethnic groups and by groups with different levels of education.

Given the differences in the smoking rates of the different subgroups and the suggestion that African American students appear to be the fastest growing subpopulation of college student smokers (11), knowledge of the possible differences in experience and perceptions may be helpful to planners of targeted tobacco control interventions.

Future research in other geographic locations may add to the body of knowledge about the products and their use. Inclusion of young adults from other racial or ethnic groups such as Asian Americans, Arab Americans, and Native Americans would provide insight into the use of NTPs in other population segments. Segmenting groups by sex would help delineate whether young men and women differ in their use and perceptions about NTPs. Young adults themselves provide valuable insight as to how nontraditional and traditional tobacco products contribute to their social lives, risk taking, health concerns and perceptions, and acceptance of future risks.

## Figures and Tables

**Table 1:** Recruitment Statistics of Participants in Focus Groups to Assess Use of Nontraditional Tobacco Products Among Adults Aged 18–22, 2002

**Characteristic**	**Chattanooga, Tennessee**		**Dallas, Texas**	
Race of focus group participants (number of groups)	White (4)	African American (4)	White (4)	Hispanic (4)
Number screened for participation in focus groups	43	40	104	47
Number (%) screened who were eligible and who agreed to participate.	36 (83)	36 (90)	34 (32)	31 (66)

**Table 2:** Nontraditional Tobacco Products Reported as Ever Used^a^ by Focus Group Participants, by Race or Ethnicity  Dallas, Texas, and Chattanooga, Tennessee, 2002

Product	Whites		Hispanics		African Americans		Total** N = 137 n (%)**

	In college** n = 35 n (%)**	Not in college** n = 35 n (%)**	In college** n = 16 n (%)**	Not in college** n = 15 n (%)**	In college** n = 18 n (%)**	Not in college** n = 18 n (%)**	
Cigars: Swisher Sweets	35 (100)	32 (91)	13 (81)	13 (87)	12 (67)	14 (78)	119 (87)
Cigars: Black & Mild	34 (97)	34 (97)	14 (88)	14 (93)	18 (100)	17 (94)	131 (96)
Herbal cigarettes	19 (54)	9 (26)	8 (50)	6 (40)	2 (11)	3 (17)	47 (34)
Bidis	10 (29)	6 (17)	5 (31)	3 (20)	5 (28)	3 (17)	32 (23)
Herbal smokeless	7 (20)	8 (23)	2 (13)	4 (27)	2 (11)	1 (6)	24 (18)
Shishas	1 (3)	0 (0)	3 (19)	1 (7)	0 (0)	0 (0)	5 (4)
Kreteks	5 (14)	0 (0)	0 (0)	0 (0)	0 (0)	0 (0)	5 (4)

a *Ever used *indicates having tried at any time any of the 6 nontraditional tobacco products chosen for the study.

## Appendix.

For this study, nontraditional tobacco products include the following:

**Cigars:** Products that contain air-cured, fermented tobacco. A Black & Mild is a small cigar that can be smoked like a cigarette with the smoke inhaled into the lungs. A Swisher Sweet is available as a thick cigar or a small, inexpensive, cigarette-like cigar (cigarillo). A Swisher Sweet can be flavored (e.g., menthol-flavored).

**Herbal cigarettes:** Vegetable-based cigarettes that manufacturers claim contain no tobacco or nicotine. They are made of herbs such as ginseng, jasmine, and catnip and may contain flavoring such as cherry, chocolate, or marshmallow. They are often sold in colorful packaging that displays playful or innocuous symbols (e.g., butterflies, leprechauns).

**Smokeless herbal chews:** Similar to chewing tobacco but with manufacturers' claims of no tobacco or nicotine. They are made from herbal ingredients such as red clover, orange peel, and bee pollen; they come in flavors such as cinnamon, wintergreen, or licorice.

**Bidis:** Small, hand-rolled, usually flavored and unfiltered tobacco cigarettes from India and southeast Asia. In the United States, they come in a variety of flavors, such as chocolate, vanilla, strawberry, root beer, or mango; they cost about half as much as regular cigarettes.

**Kreteks (clove cigarettes):** Cigarettes from Indonesia containing a mixture of tobacco leaves and clove spices. The name kretek is derived from the crackling sound that cloves make when burned.

**Shisha:** A type of tobacco used when smoking a shisha or hookah pipe. It is made from tobacco leaves mixed with fruit shavings or flavoring. It is held together by a sweet, sticky molasses. Common flavors include apple, strawberry, apricot, and (recently) cola and cappuccino.

## References

[1] Savitz DA, Meyer RE, Tanzer JM, Mirvish SS, Lewin F (2006). Public health implications of smokeless tobacco use as a harm reduction strategy. Am J Public Health.

[2] Richter PA, Pederson LL, O'Hegarty MM (2006). Young adult smoker risk perceptions of traditional cigarettes and non traditional tobacco products. Am J Health Behav.

[3] (2005). Centers for Disease Control and Prevention. Cigarette smoking among adults – United States, 2004. MMWR Morb Mortal Wkly Rep.

[4] (2005). U.S. Department of Health and Human Services. Summary of findings from the 2001 National Household Survey on Drug Use and Health.

[5] Rigotti NA, Lee JE, Wechsler H (2000). U.S. college students' use of tobacco products: results of a national survey. JAMA.

[6] (2004). Centers for Disease Control and Prevention. Cigarette use among high school students — United States, 1991-2003. MMWR Morb Mortal Wkly Rep.

[7] (2003). Centers for Disease Control and Prevention. Tobacco use among middle and high school students — United States, 2002. MMWR Morb Mortal Wkly Rep.

[8] Soldz S, Huyser DJ, Dorsey E (2003). Characteristics of users of cigars, bidis, and kreteks and the relationship to cigarette use. Prev Med.

[9] Soldz S, Huyser DJ, Dorsey E (2003). The cigar as a drug delivery device: youth use of blunts. Addiction.

[10] Soldz S, Dorsey E (2005). Youth attitudes and beliefs toward alternative tobacco products: cigars, bidis, and kreteks. Health Educ Behav.

[11] Patterson F, Lerman C, Kaufmann VG, Neuner GA, Audrain-McGovern J (2004). Cigarette smoking practices among American college students: review and future directions. J Am Coll Health.

